# Metabolic changes in vitamin D receptor knockout mice

**DOI:** 10.1371/journal.pone.0267573

**Published:** 2022-06-17

**Authors:** Sue Lynn Lau, Rebecca A. Stokes, Beverly Ng, Kim Cheng, Roderick Clifton-Bligh, Jenny E. Gunton

**Affiliations:** 1 Westmead Hospital, Sydney, New South Wales, Australia; 2 Faculty of Medicine and Health, The University of Sydney, Camperdown, New South Wales, Australia; 3 The Centre for Diabetes, Obesity and Endocrinology Research (CDOER), The Westmead Institute for Medical Research, The University of Sydney, Sydney, New South Wales, Australia; 4 Garvan Institute for Medical Research, Darlinghurst, New South Wales, Australia; Max Delbruck Centrum fur Molekulare Medizin Berlin Buch, GERMANY

## Abstract

VDR expression has been found in many cell types involved in metabolism, including the beta-cells of the pancreatic islets. Activated vitamin D and its interactions with the vitamin D receptor (VDR) are implicated in glucose homeostasis. We investigated the metabolic phenotype of the VDR-null (VDRKO) mouse at early and middle age. All offspring of heterozygote VDRKO breeding-pairs were fed ‘rescue diet’ from weaning to normalize calcium and phosphate levels in VDRKO and to avoid confounding by different diets. Glucose tolerance testing was performed at 7 and 24 weeks of age. Insulin tolerance testing, glucose-stimulated insulin secretion, body-composition studies and islet isolation were performed at 25–27 weeks. Glucose-stimulated insulin secretion was tested in isolated islets. VDRKO mice had reduced bone density, subcutaneous fat mass and muscle weights compared to WT mice. Despite reduced fat mass, glucose tolerance did not differ significantly. Male but not female VDRKO had improved insulin sensitivity. Global loss of VDR has significant effects on organs involved in energy metabolism and glucose homeostasis. In the setting of decreased fat mass, a clear effect on glucose tolerance was not present.

## Introduction

The function of the vitamin D system in the pancreatic beta-cell and its wider role in glucose tolerance and whole-body metabolism in humans is unclear [1]. With recognition of widespread vitamin D insufficiency in today’s relatively sun-averse society and rising rates of diabetes and obesity worldwide, the question of whether vitamin D may play a role in diabetes is of interest at both population health and molecular levels.

However, the question has proven complex. While epidemiological studies mostly demonstrate inverse associations between vitamin D status and diabetes risk, trials of vitamin D supplementation show variable results [1, 2]. The active hormonal form of vitamin D, 1,25-dihydroxyvitamin D3, interacts with the vitamin D receptor (VDR) to regulate gene transcription in a range of metabolically active tissues, including beta-cells [3, 4]. How this translates into *in-vivo* and clinical outcomes is not clear.

VDR null or knockout (VDRKO) mice have been generated by four independent groups in Tokyo [5], Boston [6], Leuven [7] and Munich [8]. The mice lack either the first or second zinc finger in the DNA-binding domain and are fully resistant to the actions of vitamin D on the VDR. They develop growth retardation, hypocalcaemia, hypophosphataemia and rickets after weaning. Whilst possessing a full coat at birth, they develop progressive alopecia throughout life. To rescue the bone and mineral phenotype, the VDRKO “rescue diet” was developed and it normalizes serum calcium and phosphate levels [9]. Alopecia is unaffected by the diet.

The Erben VDRKO mouse (Munich) has elevated blood glucose levels after oral or subcutaneous glucose administration and reduced maximal serum insulin levels compared to wild-type (WT) controls [10]. In contrast, the Demay VDRKO mice (Boston) showed no difference in glucose tolerance between knockouts and wild-types at 8 weeks of age, despite significant changes in expression of renin-angiotensin-aldosterone system components observed in their islets [11]. The Erben mice express a VDR with intact hormone-binding but no transcription factor activity due to defective DNA-binding. Vangoitsenhoven et al compared glucose tolerance in the Tokyo VDRKO null mice with both WT mice and VDRΔAF2 mutants (developed from the same VDRKO strain), which express VDR incapable of mediating transcriptional activation [12]. Unlike VDRKO, VDRΔAF2 mutants do not develop alopecia. In those mice, there were no differences in glucose tolerance, insulin tolerance, glucose-stimulated insulin secretion (GSIS) or pancreatic insulin content between VDRΔAF2, VDRKO or WT at 12 or 24 weeks, noting however that both mutants had lower body fat and bone mineral content than WT.

In this study, we explored the metabolic phenotype of the Demay VDRKO mouse at early and later ages in both genders.

## Results

**Fig 1:**
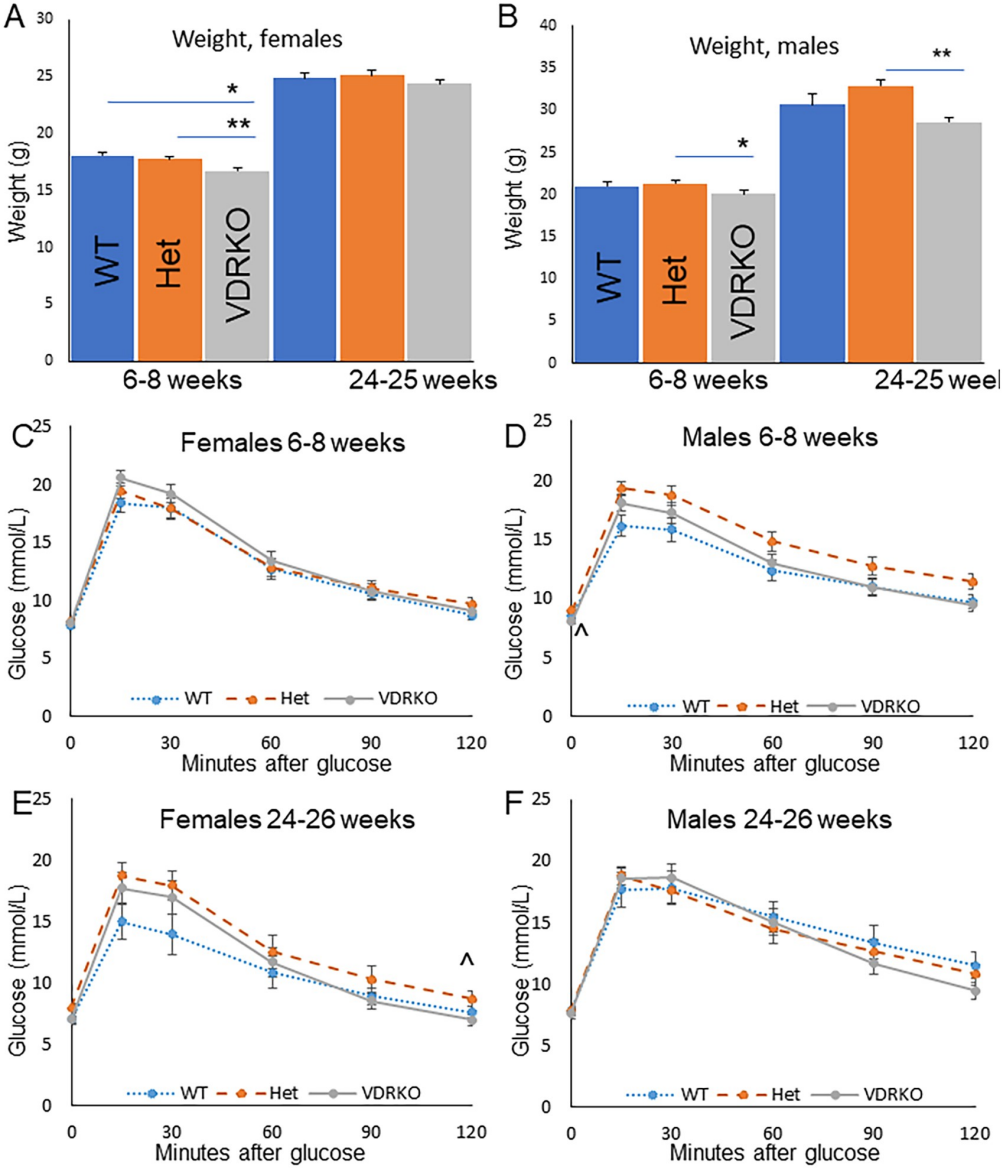
Weight and glucose tolerance in mice eating rescue diet. A) Body weight in female mice. B) Body weight in male mice. C) Glucose tolerance in Females at 6–8 weeks, D) Glucose tolerance in male mice at 6–8 weeks. E) glucose tolerance in older female mice. F) glucose tolerance in older male mice. For young females, N = 34 WT, N = 51 Het, N = 53 VDRKO. For young males, N = 32, 42 and 43. For older females and males N = 15 WT, 22 hets and 23 VDRKO per gender. *p<0.05 **p<0.01, ***p<0.001 for VDRKO versus WT. ^ p<0.05 Het versus VDRKO. Data shows mean ± SEM, p-values corrected for multiple comparisons.

Young female vitamin D receptor knockout mice (VDRKO) were significantly lighter than their wild-type (WT) and heterozygous VDR-knockout (Het) littermate comparators at 16.7±0.2g versus 18.0±0.3g in WT and 17.7±0.2g in Het (p<0.001 and p<0.01 respectively versus VDRKO, Fig 1A). These differences had resolved by 24–26 weeks of age, noting smaller numbers of mice. Male Het mice were heavier than VDRKO males at both age groups (Fig 1B).

### Glucose tolerance in young and old female and male mice

At 6–8 weeks of age, in female mice, glucose tolerance tests were similar in the different genotypes (Fig 1C, p = ns by ANOVArm). At 6–8 weeks male Het mice had slightly worse fasting glucose than VDRKO (Fig 1D). By 24–26 weeks, glucose tolerance had improved in the WT females, but there were no significant differences between genotypes (Fig 1E, p = ns by ANOVArm). Male mice had no significant differences in glucose tolerance overall or at any timepoint when 24–26 weeks old (Fig 1F, p = ns by ANOVArm).

**Fig 2:**
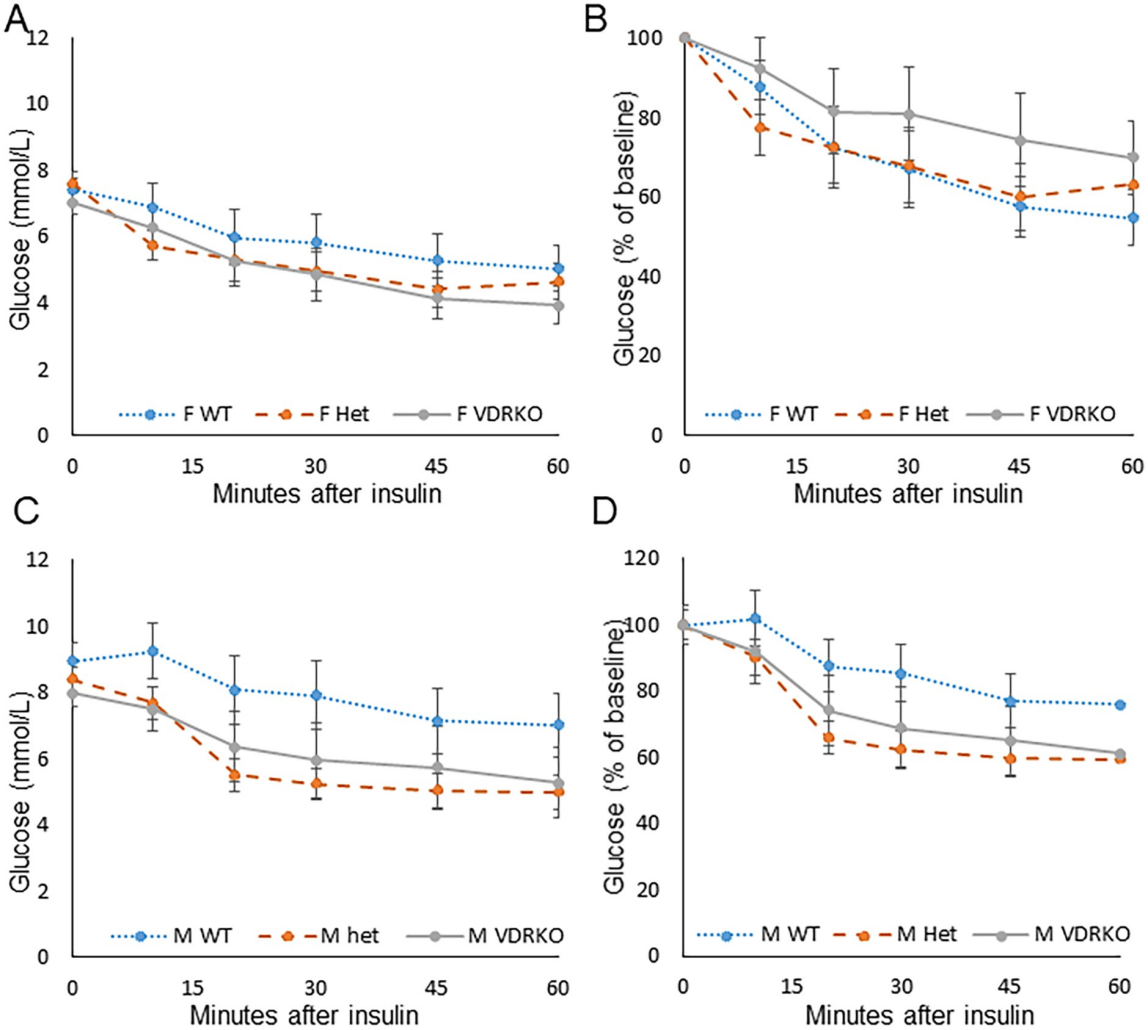
Insulin sensitivity at 25–26 weeks, assessed by glucose response to insulin. A) Female glucose levels after insulin administration. B) Female results as % of baseline glucose for the individual mouse. C) Male glucose results after insulin injection. D) Male results as % of baseline glucose values. Data shows mean ± SEM. All mice were fed rescue diet. N≥14 mice per group.

Insulin sensitivity was assessed at 26 weeks by insulin tolerance testing. There were no significant differences between WT and VDRKO mice, including by ANOVArm. There were no differences in insulin tolerance in females, including by ANOVArm, shown in Fig 2A as absolute glucose and Fig 2B as percentage of basal glucose. Male Het mice had a tendency to larger decreases in glucose following insulin treatment than WT mice, but this was not significant on testing by ANOVArm, shown as absolute glucose (Fig 2C) and percent of basal glucose (Fig 2D).

### Glucose-stimulated insulin secretion at 25 weeks

**Fig 3:**
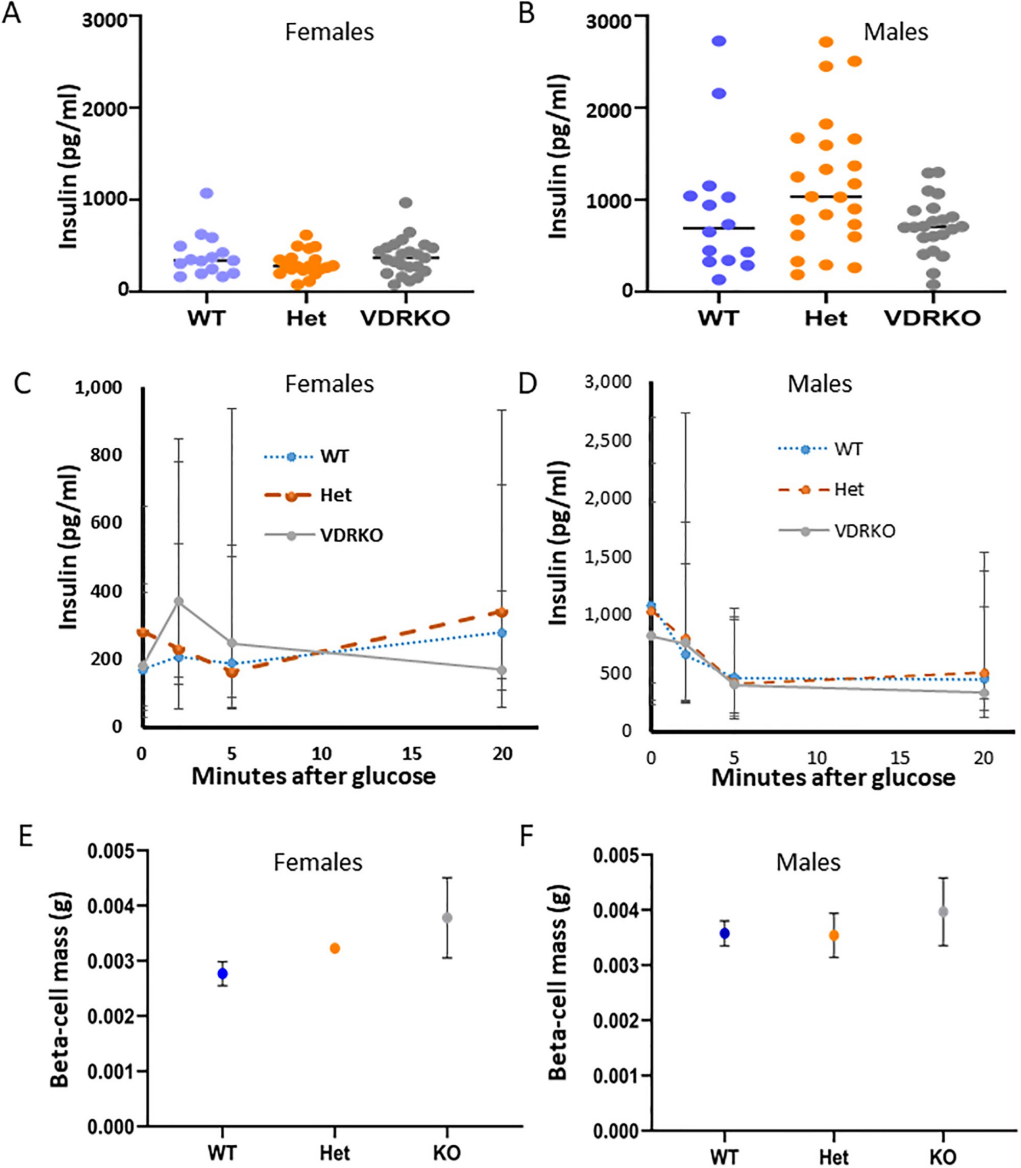
Fasting insulin and glucose stimulated insulin secretion in mice at 26 weeks of age. A) Female fasting insulin. B) Male fasting insulin. C) Female glucose stimulated insulin secretion. D) Male glucose stimulated insulin secretion. E) Beta cell mass in female mice. F) Beta cell mass in male mice. A and B show data for each mouse. C and D show median and interquartile ranges. N≥14 mice per group for figures A-D. Figures E and F show mean and SEM. N = 3 WT, 4 Het and 5 VDRKO females, and N = 4 WT, 5 Het and 9 VDRKO males for beta cell mass.

At baseline, there were no significant differences in fasting insulin between genotypes in females (Fig 3A). Levels of fasting insulin were higher in males than females, and showed a trend to being higher in male heterozygotes which did not achieve statistical significance (Fig 3B). Most mice at this age had poor first phase insulin secretion (<10 minutes from glucose load), without significant differences between genotypes in either females (Fig 3C) or males (Fig 3D). There were also no significant differences in early second phase insulin secretion, assessed at 20 minutes, with wide ranges (Fig 3).

### Size and body composition

**Fig 4:**
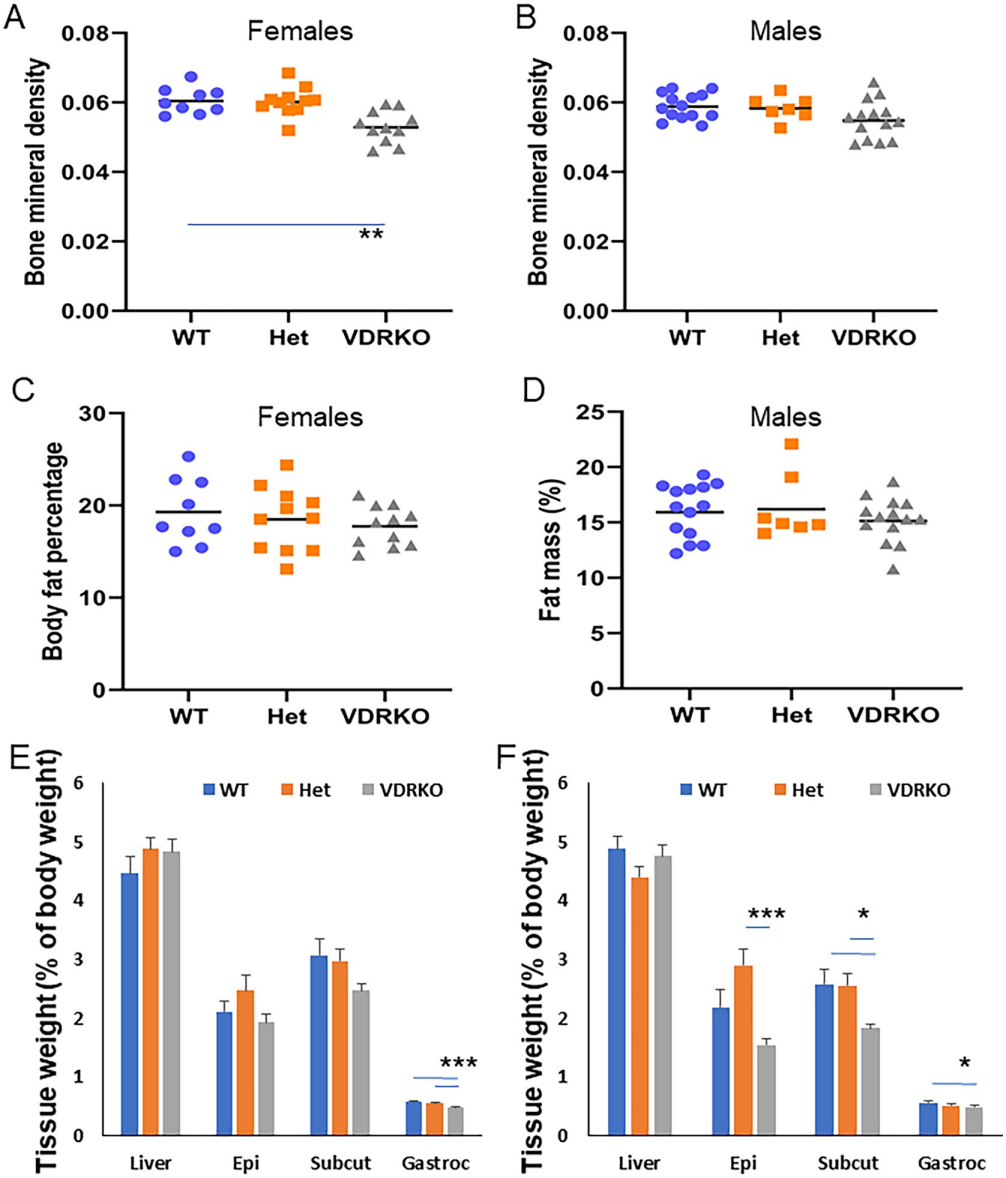
Body composition measures. A) Bone mineral density in female mice. B) BMD in male mice. C). Body fat percentage measured by DEXA in female mice. D) Body fat percentage in male mice. E) Tissue weights in female mice, expressed as percent of body weight. F) Tissue weights in male mice. Epi = epigonadal fat. Subcut = subcutaneous fat. Gastroc = gastrocnemius. * = p<0.05, ** p<0.01 and *** = p<0.001 for the indicated comparison. All p-values are corrected for multiple comparison (Bonferroni). N≥9 mice per group.

Body composition was assessed at 27 weeks by DEXA in a subset of 6–13 mice per group. There were no significant differences in body weight between KO and WT in these smaller groups. DEXA revealed lower average bone mineral density in VDRKO females compared to either Het or WT mice (Fig 4A, p<0.01 for both). Bone mineral density tended to be lower in male VDRKO (p = 0.077 versus WT, Fig 4B). VDRKO mice had numerically lower body fat percentages, although this was not statistically significant (Fig 4C and 4D).

Snout-tail length was significantly shorter in VDRKO females compared to WTs or Hets (VDRKO 10.3cm vs 10.6cm WT and 10.6cm Hets, p = 0.01 for both), and in VDRKO males versus WT (VDRKO 10.5cm vs 10.8cm in WT, p = 0.01) but VDRKO were not significantly shorter than Hets (10.7cm p = 0.077).

Liver weight did not differ between WT and VDRKO in females (Fig 4E) or males (Fig 4F). Muscle weights are lower in VDRKO mice (expressed as percentage of total body weight) with gastrocnemius significantly lighter in both genders (Females: 0.49% vs. 0.58% of body weight, p<0.01. Males: 0.50% vs. 0.57%, p = 0.01). We have previously reported in a different cohort of mice that quadriceps weight is lower in VDRKO than WT mice [9]. Subcutaneous fat mass was also significantly lighter in VDRKOs in body females (Fig 4E) and males (Fig 4F) and there was a trend towards lower epigonadal fat mass in male VDRKOs (1.55% vs. 2.19% p = 0.064).

### Ex-vivo glucose-stimulated insulin secretion (GSIS) in female islets

**Fig 5:**
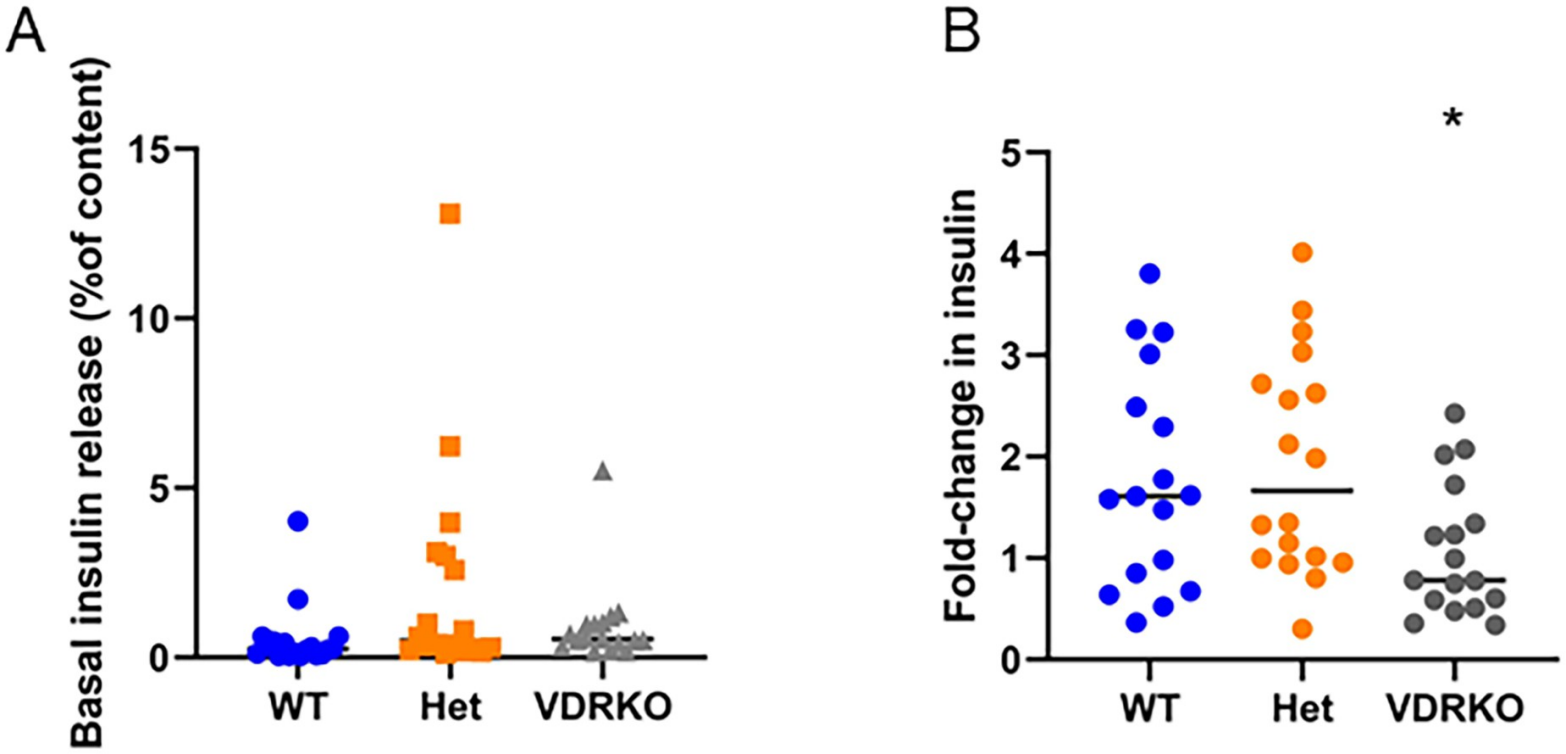
Insulin release from isolated islets in females. A) Low glucose (1mM) insulin release expressed as a percentage of total insulin content. B) Fold-increase with 25mM glucose compared to 1mM glucose for each set of islets. * p<0.05 for VDRKO versus WT, non-parametric testing. N≥12 per group.

VDRKO mice have altered size and body composition. To test whether there were effects of VDR deletion on islet function ex vivo, islets were isolated from female mice and GSIS was tested. There was no difference in basal insulin secretion at 1mM glucose concentration in females (Fig 5, left panel).

Islets from female VDRKO had a diminished insulin secretory response at 25mM versus 1mM glucose (expressed as fold-change) compared to WT islets (Fig 5, right hand panel), but this was not significant after correction for multiple comparisons (p<0.08).

Vitamin D receptor knockout mice require ‘rescue diet’ to remain healthy. This is high in calcium, phosphate and lactulose. We have previously reported that this normalizes serum calcium and phosphate in VDRKO mice compared to their littermate controls [9]. The diet is therefore quite different from normal mouse chow. We tested whether rescue diet was associated with changes in glucose tolerance in WT and Het mice. VDRKO mice cannot be tested as the mice in our line VDRKO do not survive on chow. WT and Het mice were fed rescue or normal chow diet from weaning and glucose tolerance was compared.

**Fig 6:**
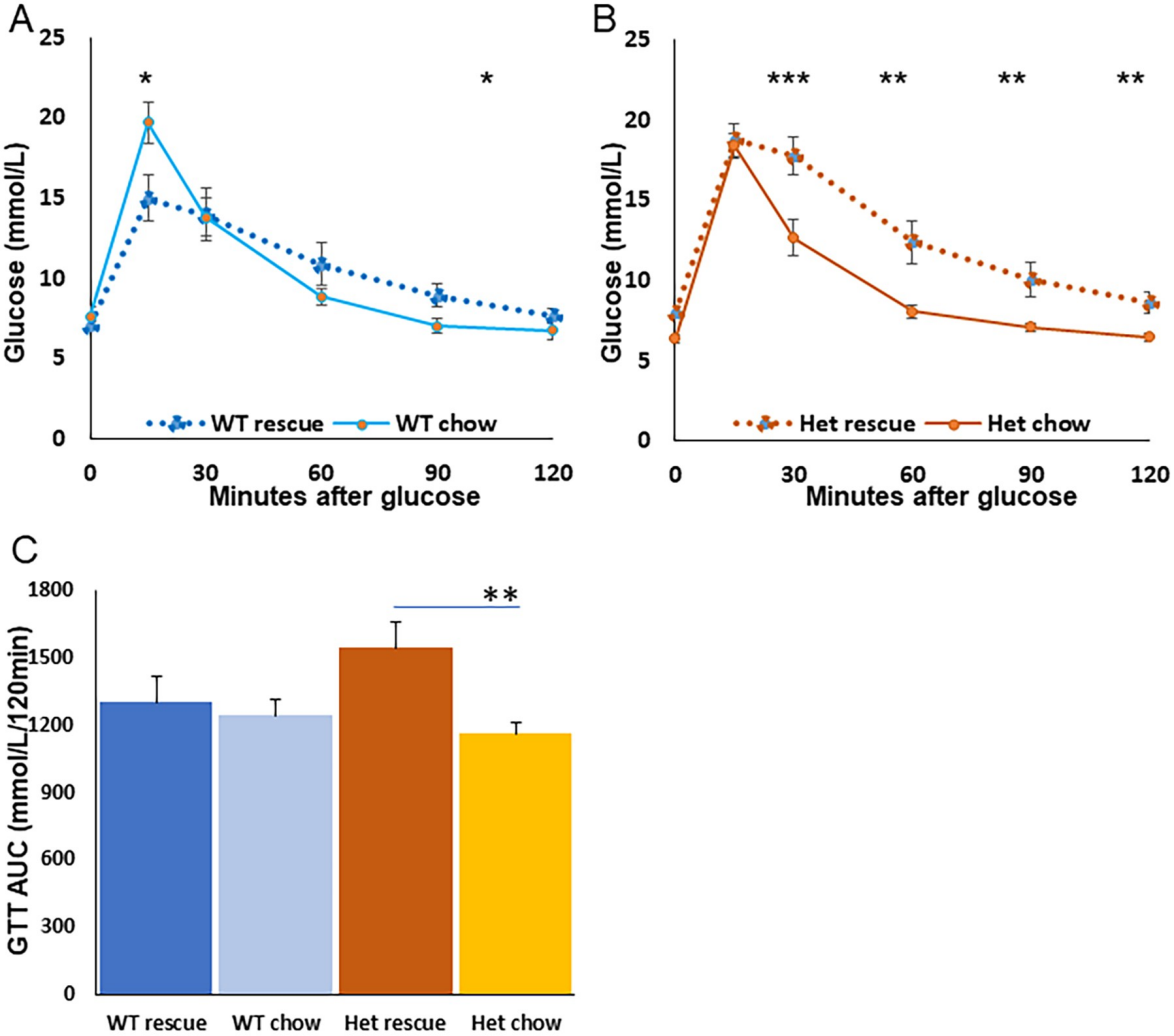
Glucose response to rescue diet compared to chow in WT and Het mice. A) Glucose tolerance test in WT mice. B) GTT in Het mice. C) AUC of GTTs. All mice are female. WT chow N = 4, WT rescue diet N = 8. Het chow N = 8 Het rescue N = 9.* p<0.05, ** p<0.01. GTT AUC = glucose tolerance test area under the curve.

Unexpectedly, the glucose tolerance response to diet differed between WT and Het mice. WT mice had a rise in peak glucose, and a fall in glucose later in the GTT (Fig 6A). However, Het mice had significant deterioration in glucose tolerance on rescue diet, compared to chow and glucose had not returned to baseline at 2 hours (Fig 6B). Fig 5C shows the area-under-the-curve (AUC) for GTT in the 4 groups of mice. Heterozygotes had significantly worse AUC on rescue diet with a 33% deterioration (Fig 6C), whereas WT mice did not have any significant change in glucose tolerance.

## Discussion

Consistent with studies in 8-week old Demay VDRKO mice [13] and the Tokyo VDRKO and VDRΔAF2 mutants [12], but in contrast to Zeitz’s work on the Munich VDRKO mouse [10], we found no significant difference in glucose tolerance of Demay VDRKO mice on a C57Bl/6 background at 7 or 24 weeks of age. There were also no differences in insulin sensitivity. However, *ex-vivo* islet studies did suggest decreased β-cell function in female VDRKOs.

Contrary to other studies in this VDRKO mutant [14], we did not find a difference in overall bodyweight at 24 weeks of age. However, differences in body composition in both genders in our study were consistent with observations across other VDRKO studies, specifically, decreased bone density, and lower subcutaneous adiposity and muscle weights. As bone, muscle and fat all potentially influence β-cell function and/or insulin sensitivity, similarity in glucose tolerance observed between WT and VDRKO mice does not exclude a role of VDR in glucose homeostasis, but may be the net outcome of divergent effects on different organ systems. The lean phenotype of global VDRKO mice has previously been described and attributed to increased expression of uncoupling protein-1 in white adipose tissue, resulting in increased energy expenditure [14]. Observation of lean phenotype in VDRΔAF2 mutants that have normal fur covering suggests that this metabolic phenotype is not merely due to alopecia [12].

Despite decreased adiposity, Vangoitsenhoven et al found no difference in insulin sensitivity in the VDRKO or VDRΔAF2 mutants at 12 weeks of age. Glucose tolerance did not differ at 24 weeks and the ITT was not repeated, so direct comparison to our study cannot be made, but the results are similar. Interestingly, VDR knockout specifically in murine endothelial cells increased insulin sensitivity and lowered blood glucose levels after glucose challenge at 24 weeks of age [15]. Reduced insulin secretion in isolated islets was thought secondary to improved insulin sensitivity. In contrast, mice with macrophage-specific deletion of VDR demonstrated an insulin resistant phenotype [16]. These studies highlight how the metabolic phenotype of whole body VDRKO may result from a complex interplay of effects at different tissue sites.

The already complex state of VDR mice is further complicated by the different effects of rescue diet, with heterozygous mice showing significant deterioration in glucose tolerance on rescue diet, but wild-type mice being protected from that issue. It is not possible to test the effect of chow diet in our strain of VDRKO line as mice rapidly become very unwell and die.

To clarify the role of VDR in insulin secretion, Morro et al examined the effect of β-cell VDR overexpression in transgenic mice [17]. These mice had normal islet cell mass at baseline but were protected from chemically-induced diabetes, with reduced loss of islet cell area and insulin secretion. There is strong evidence that VDR modulates inflammation and β-cell survival through association with BAF complexes [18]. Interestingly, VDRKO mice bred on a CD1 background were relatively protected from glucose intolerance during high-fat feeding, despite lower insulin levels. This suggests that improved insulin sensitivity accompanying the lean phenotype may counteract any adverse effect on beta-cell survival during inflammatory stress in this VDRKO model [14].

Our *ex-vivo* islet studies suggest impaired insulin secretion in VDRKO β-cells, though there was a high degree of variability between mice. Results fit with other work demonstrating enhancement of GSIS in response to calcitriol, the active hormonal ligand of VDR [19]. Loss of this effect in islets isolated from Demay VDRKO mice fed vitamin D3 supplemented standard rodent chow confirms that the VDR is implicated in insulin secretion.

In summary, VDRKO mice are lean, and when eating ‘rescue diet’ which normalizes their blood calcium and phosphate, have normal glucose tolerance. Insulin tolerance testing did not show significantly altered insulin sensitivity at 6 months of age in VDRKO. In vivo GSIS was also not different. However, isolated islets from female VDRKO mice showed significantly lower insulin release after glucose challenge suggesting that there may be subtle deficits in beta-cell function which are masked in vivo.

Interestingly, the rescue diet, which is needed for survival in VDRKO mice, has divergent effects on glucose tolerance depending on whether mice are wild-type or heterozygous for normal VDR. Because of the many confounders in vivo, studies in beta-cell specific VDR null mice may be needed to elucidate effects of VDR in beta-cells.

## Materials and methods

### Mouse breeding, housing, diets and care

Mouse-breeding was conducted at Australian BioResources, Moss Vale. Experimental mice were issued to the Biological Testing Facility at the Garvan Institute of Medical Research where they were exposed to a 12-hour light/dark cycle (0700–1900 on, 1900–0700 off) and housed in standard filtered boxes with free access to autoclaved water and food. According to the experimental protocol, mice were fed VDRKO rescue diet (2% calcium, 1.25% phosphorus and 20% lactose) custom manufactured by Glenn Forest Stockfeeders, Perth. The study was conducted according to the guidelines of Animal Research Act 1985 and the “Australian Code for the care and use of animals for scientific purposes” and the ethics application was approved by the St Vincent’s/Garvan Animal Ethics Committee.

Heterozygous mice from the Demay VDRKO line, previously described [6, 11], were used for breeding. The mice are on an inbred C57Bl/6 background. All heterozygote breeding pairs were fed standard rodent chow. Their wild-type and knockout offspring formed the experimental litters. All offspring of heterozygote pairs, regardless of genotype, were fed rescue diet from weaning.

### Experimental timeline

At 7 weeks, VDRKO and WT mice underwent a first glucose tolerance test. After maturation to 24 weeks of age, a second glucose tolerance test was performed, followed 1 week later by glucose-stimulated insulin secretion, a week later by insulin tolerance testing and then sacrifice. A sub-set of mice underwent DEXA scanning prior to sacrifice. At sacrifice, mice were weighed and the pancreas collected for islet cell isolation and *ex vivo* insulin secretion studies. Organ weights were measured post-sacrifice.

### Experimental techniques

#### Glucose tolerance testing (GTT)

Mice were weighed on the morning of testing, fasted for 4–6 hours and tested between 3–5 pm that afternoon. Blood was collected for glucose measurement via tail nick at baseline (time 0) and at 15, 30, 60, 90 and 120 minutes after an intraperitoneal injection of 20% glucose at a dose of 2g/kg per mouse. Glucose concentration was quantified using an Abbott FreeStyle Papillon Mini glucometer.

#### Glucose-stimulated insulin secretion (GSIS)

After a 4–6 hour fast, 20% glucose was injected intraperitoneally at a dose of 3g/kg per mouse. 20uL of whole blood was expressed from tail-nick into a 1.5ml Eppendorf containing 2 μL of EDTA/proteinase inhibitor cocktail (10ml of 0.5M EDTA, 1 proteinase inhibitor cocktail tablet, complete Mini, Roche Diagnostics, USA) immediately prior to, and at 2, 5 and 20 minutes following glucose injection. After immediate centrifugation, the supernatant was aspirated and briefly kept on ice before storage at -20°C whilst awaiting insulin ELISA assay.

#### Insulin tolerance test (ITT)

After a 4–6 hour fast, 0.5 units/kg of insulin (Actrapid 100 IU/ml, Novo Nordisk) was diluted in 1x PBS with 1% bovine serum albumin and injected intraperitoneally. Blood glucose concentrations were measured at baseline (0 min) and at 10, 20, 30, 45 and 60 minutes following injection. If the BGL fell below 2 mmol/L, the mouse was given an intraperitoneal injection of 20% glucose and the test was terminated. The lowest blood glucose recorded was used as the value for the remaining time-points.

#### Dual-energy x-ray absorptiometry (DXA scanning)

Mice were weighed and anaesthetized using 2,2,2-tribromoethanol (Avertin) via intraperitoneal injection. Mice were placed in a prone position, with four legs extended and taped to the scanning platform. DXA scanning was performed using a Lunar PIXImus2 machine (GE, USA). After excluding the head and feet from the region of interest, data was analysed for bone mineral density, bone mineral content, lean body mass, fat mass and fat percentage using PIXImus software.

#### Sacrifice and tissue collection

Mice were sacrificed in the non-fasted state using 2,2,2-tribromoethanol injected intraperitoneally to achieve deep sedation followed by cardiac puncture and exsanguination. After sacrifice, animals were stretched and measured from snout to tail-base. The following organs were resected and weighed, with results expressed as percentage of total bodyweight. Beta cell mass was calculated as we have previously reported, measuring beta-cell area in separated pancreatic sections as a percentage of pancreatic area on that slide, and multiplying by the pancreatic weight [20].

#### Mouse islet isolation

After deep sedation with 2,2,2-tribromoethanol and exsanguination, the Ampulla of Vater was clamped and 3ml of 0.25mg/mL Liberase-Enzyme Blend-RI (Roche, Indianapolis, IN) diluted in M199 was injected via a 30-gauge needle into the common bile duct. Distension of the entire pancreas was observed. Each pancreas was removed, placed in a 50ml falcon tube and digested in a 37°C water bath for 16.5 min. Digestion was terminated with the addition of cold M199 with 10% FCS. 2 wash steps with M199 + 10% FCS were then performed, combined with vigorous vortexing and shaking of the digested pancreas, prior to filtering through a 425-micron sieve (US standard sieve series, A.S.T.E. E-11 specifications dual MFG, Co. Chicago, Il) to remove larger non-islet material. The filtered material was centrifuged for 2 minutes and the supernatant decanted. A gradient was formed with 20ml Ficoll-Plaque Plus (Amersham) 1.077 and 10 ml M199 alone and then centrifuged at 2830 rpm for 22 minutes, allowing the islets to separate into the interface between the two layers. Islets were pipetted from this layer into a clean tube, replenished with M199 + 10% FCS and centrifuged at 1250rpm for 2 min. A further two wash steps were performed before a final centrifugation step. Islets were then pipetted into a petri dish and visualised under the microscope. Islets were handpicked by pipette into a 1.5ml Eppendorf tube. Care was taken to avoid selecting as much non-islet/acinar material as possible.

Ex-vivo GSIS For each mouse, 3 sets of 12–15 islets were hand-picked after isolation and placed in Eppendorf tubes containing 1 ml of 37°C, serum-free, 1mM glucose DMEM at pH 7.4. Holes were made in the lid to allow adequate oxygenation whilst they were kept at 37°C in a 0.5% CO2, 21% O2 incubator. After 2 hours, Eppendorf tubes were washed with warm 1mM glucose DMEM in a 37°C water bath. At time zero, the media was replaced with 1 ml of fresh, warm DMEM with 1mM glucose. After 15 min, Eppendorf tubes were removed, inverted 3 times and then centrifuged briefly. 10μL was pipetted from the supernatant and diluted in 490μL of 1%BSA-PBS. 2 samples were taken from each tube. The supernatant was then aspirated and 1mL of warmed 25mM glucose DMEM was added to the islet pellet. After a further 15 min in the 37°C water bath, supernatant samples were obtained and diluted in a similar manner. The supernatant was removed again, 500mL of RIPA buffer added to lyse the remaining islet pellet and the sample stored for later assay of total insulin content. Samples were all stored at -20°C until the insulin ELISA was performed.

For each set of islets, the average insulin concentration of the 2 samples collected was obtained. If there was a wide discrepancy the data from that set of islets was excluded. Values at low and high glucose concentrations were expressed as a percentage of the total insulin content of the islets, calculated from the RIPA buffer sample. The fold change of insulin secretion at high versus low glucose concentrations was calculated and averaged for the 3 sets of islets per mouse.

The Crystal Chem Rat insulin ELISA kit (Chicago USA) was used to determine the insulin concentrations of mouse plasma samples and islet supernatant samples.

#### Statistical analysis

Comparison of the means of 2 independent groups was performed using Student’s t-test, wherever variables approximated a normal distribution. Analyses were two-tailed and a p-value of <0.05 was considered significant. Comparisons of 3 groups were corrected for multiple comparisons, using Bonferroni correction. Where specified, some variables were log-transformed to better approximate normality, though for the purposes of the Figs, the non-log-transformed values have been graphed on the y-axis. Overall GTT and ITT results were assessed by ANOVA for repeated measures (ANOVArm) in SPSS. Unless otherwise specified, data shows mean ± SEM.

For non-normally distributed variables, non-parametric testing was performed using the test specified in the text. In such cases, the p-values indicated are for comparison of the group medians, though for consistency, graphs continue to represent the mean values on the y-axis. Statistical analysis was performed using Excel or PASW statistics 18.0.
